# Fisher Information Limits on Genetic Stability, Diversity, and Regulation

**DOI:** 10.1007/s11538-026-01720-5

**Published:** 2026-08-05

**Authors:** J. S. Glasenapp, R. A. Gatenby

**Affiliations:** Integrated Mathematical Oncology Department, Cancer Biology and Evolution Program, Moffitt Cancer Center, 12902 Magnolia Dr, 33612 Tampa, FL USA

**Keywords:** Information theory, Genetics, Mutations, Evolution, Error limits

## Abstract

Life and evolution require precise yet imperfect transmission of genetic information across generations. Accurate transmission maintains the genetic blueprint for phenotypes that succeed in current conditions, while some transmission error is necessary to generate heritable diversity that can further increase fitness and hedge against future environmental change. This background noise, however, risks excessive mutation accumulation that degrades fitness (“Muller’s ratchet”) or even destroys genetic information beyond recovery (“error catastrophe”). Across the history of life these competing pressures resolve into two regimes: during environmental fluctuation, a higher mutation rate aids survival; during prolonged stability, populations converge toward an evolutionarily stable state (ESS) in which mutations can only reduce fitness. We model this tension with a Fisher Information framework in which genetic inheritance is a noisy two-state channel. The nontrivial eigenvalue of the channel’s symmetric circulant transition operator governs the decay of allelic contrast across generations; selection modifies this eigenvalue by suppressing the channel’s switching (mutation) probability, acting as an anti-depolarizing force. We show this compensation is bounded: an instability boundary at p(1-p)=1/9 marks the point beyond which selection can no longer maintain informational stability — a boundary that coincides with the loss of quantum coherence in the formally equivalent depolarizing channel and with thermodynamic fine-graining failure, suggesting that genetic stability, regulatory fidelity, and structural order share a common information-geometric limit. We apply this framework to cancer, framing carcinogenesis as an informational transition: a shift from host-regulated, high-fidelity transmission that maintains tissue homeostasis to a regime in which cells retain only the genetic and epigenetic changes that maximize their own proliferation — including loss of functions that serve the host and gain of functions that improve competition for space and nutrients and evasion of the predator-like immune response. Because cancer cells operate farther from this information-geometric limit than the normal cells around them, we propose that they may pursue an informational “niche construction” strategy: producing a mutagenic, acidic, hypoxic microenvironment that pushes neighboring host cells past the same collapse boundary, driving loss of function (e.g., “T cell exhaustion”) in infiltrating immune cells. We present empirical support for this framework together with specific, experimentally testable predictions.

## Introduction

Living systems use inherited genetic information to maintain a stable, low-entropy (highly ordered) state while far from thermodynamic equilibrium (Schrodinger 1956; Gatenby and Frieden 2007). Failure to maintain the non-equilibrium condition results in death and permits evolutionary selection (Gatenby and Frieden 2007). This deep connection between information and thermodynamics represents life’s first principles and permits the evolutionary dynamics that govern the history of life.

The principles of evolution are often framed through Darwin’s fundamental postulates (Dimijian 2012): (1) Populations contain phenotypic variability; (2) these traits are heritable; (3) environmental constraints limit proliferation so that more offspring are produced than can survive, producing a “struggle for existence”. Heritable phenotypic variability in postulates 1 and 2 determines the outcomes of the struggle described in postulate 3.

The requirements for evolution confer divergent constraints on the transmission of genetic information. Since an organism’s replication is directly related to its fitness within current environmental conditions, its genetic information must be transmitted with high fidelity to ensure the success of its offspring. On the other hand, evolution requires some heritable variability within a population so that the population can generate phenotypic properties that increase fitness within current conditions and as a hedging strategy to permit population survival should the environment change.

However, any error rate in the transmission of genetic information, while necessary for evolution, comes with some hazard. In a constant environment, evolutionarily successful organisms typically reach a maximal fitness termed the Evolutionarily Stable State (ESS) (Apaloo et al. 2009), so that no mutation can increase fitness and thus all mutations are either neutral or decrease fitness (Haigh 1978; Rispe and Moran 2000). This risks progressive degradation of the information content of the genome, leading to reduced fitness (“Muller’s ratchet” Howe and Denver 2008) and even an “error catastrophe” (Eigen 2002) in which life cannot be sustained.

Thus, the history of life on Earth reflects a balance between these two risks. In periods of environmental fluctuation, the transmission of genomic information must include sufficient mutations to generate heterogeneity that will explore possible fitness gains in the current environment and allow some individuals to survive sudden changes in environmental selection forces. In contrast, during periods of prolonged environmental stability a persistently high mutation rate risks degradation of genomic information and population decline, including possible extinction (Dimijian 2012; Wei et al. 2022; Lynch et al. 2023).

The multibillion-year arc of life on Earth through large catastrophic events and prolonged stability, as well as prolonged propagation of unicellular prokaryotes and eukaryotes in culture conditions, clearly demonstrates that neither of these worst-case scenarios is common.

Here we investigate these complex dynamics using Fisher Information (FI), which quantifies the amount of information that an observable random variable carries about an unknown parameter of its underlying probability distribution (Cramér 1946; Rao 1945). In genetics, FI measures how precisely an ancestral allele frequency can be inferred from observations in subsequent generations. High FI corresponds to high inferential precision and therefore reflects robust and reliable genetic transmission.

This interpretation is formalized by the Cramér–Rao lower bound. For any unbiased estimator $\hat{\theta }$ of a parameter θ,

1 $$\begin{aligned} \textrm{Var}(\hat{\theta }) \ge \frac{1}{I(\theta )}. \end{aligned}$$

Thus, Fisher Information sets a fundamental limit on estimation accuracy. In a biological context, it provides a natural metric for assessing the fidelity of inheritance, the stability of gene regulation, and the limits of evolutionary inference.

## Genetic Transmission as an Information Channel

Genetic transmission from a parental population (source) to an offspring population (receiver) is modelled as an information channel subject to noise arising from mutation or recombination. These informational dynamics are subject to evolutionary control, which in effect opens or closes the channel: the channel is opened if the organism’s fitness allows it to proliferate, and remains closed if it dies or remains alive but non-proliferative — a state of uncertainty resembling a quantum wave function.

Furthermore, we view the mutation rate (channel noise) as a variable subject to local environmental conditions and evolutionary selection. Stressful environments such as increased acidity (Xiao 2003), heat (Kantidze et al. 2016), and inflammation (Kay et al. 2019) for mammalian cells increase the probability of DNA damage, thus increasing the mutation rate. The organism can modulate these effects by regulating the expression of DNA repair pathways (Chatterjee and Walker 2017). In highly stressful conditions that induce widespread population loss, evolutionary adaptation can favour a high mutation rate to rapidly generate phenotypic diversity, increasing the probability of an adaptation that will permit survival (Wei et al. 2022). In contrast, during periods of environmental stability in which the population phenotype has reached the ESS (Apaloo et al. 2009), mutations can only decrease fitness, resulting in evolutionary selection for a decreased mutation rate.

### Mathematical Setup

Let *p* denote the frequency of allele *A* in the source population. The source distribution is

2 $$\begin{aligned} P_X = (p,\;1-p). \end{aligned}$$

Transmission is governed by a symmetric circulant transition matrix

3 $$\begin{aligned} C = \begin{pmatrix} a & b \\ b & a \end{pmatrix}, \qquad a+b=1, \end{aligned}$$

where *a* represents faithful transmission and *b* the probability of allelic switching.

The eigenvalues of *C* are

4 $$\begin{aligned} \lambda _1 = 1, \qquad \lambda _2 = a - b, \end{aligned}$$

corresponding, respectively, to conservation of total probability and decay of allele-frequency contrast.

The offspring distribution $P_Y = C P_X$ satisfies

5 $$\begin{aligned} P_Y(A \mid p)&= ap + b(1-p), \end{aligned}$$

6 $$\begin{aligned} P_Y(a \mid p)&= bp + a(1-p). \end{aligned}$$

### General Fisher Information of the Two-State Channel

We now derive, from the definition of Fisher Information, how the eigenvalue $\lambda _2$ governs the inferential precision retained after one generation of transmission through the channel of Eq. (3). This derivation yields Eq. (10) directly, and Eqs. (14) and (13) follow from it as special cases in Sect. 3.

For a discrete distribution $P(x\mid \theta )$ over a finite outcome set, the Fisher Information carried by a single observation about the parameter θ is

7 $$\begin{aligned} I(\theta ) = \sum _{x} \frac{1}{P(x\mid \theta )} \left( \frac{\partial P(x\mid \theta )}{\partial \theta }\right) ^{2}. \end{aligned}$$

*Source Fisher Information.* Applying Eq. (7) to the source distribution itself (i.e. before any channel noise is introduced), with θ=p and outcomes $x\in \{A,a\}$ given by $P_X$ in Eq. (2), gives $\partial P(A\mid p)/\partial p = 1$ and $\partial P(a\mid p)/\partial p = -1$, so that

8 $$\begin{aligned} J(p) = \frac{1}{p}(1)^{2} + \frac{1}{1-p}(-1)^{2} = \frac{1}{p(1-p)}. \end{aligned}$$

This is the source Fisher Information quoted below as Eq. (14); it is the Fisher Information carried by direct observation of an individual’s allele, with no intervening transmission step.

*Channel output Fisher Information.* We now apply the same calculation to the offspring distribution $P_Y$ rather than the source distribution $P_X$, so that *I*(*p*) measures how precisely the *parental* frequency *p* can be inferred from an *offspring* observation after one generation of noisy transmission. Differentiating Eqs. (5)–(6) with respect to *p* gives

9 $$\begin{aligned} \frac{\partial P_Y(A \mid p)}{\partial p} = a-b = \lambda _2, \qquad \frac{\partial P_Y(a \mid p)}{\partial p} = b-a = -\lambda _2, \end{aligned}$$

using the eigenvalue identity of Eq. (4). Substituting Eq. (9) into Eq. (7) with θ=p gives the Fisher Information of the channel output for a general two-state channel with eigenvalues $\lambda _1=1$ and $\lambda _2$:

10 $$\begin{aligned} I(p) = \frac{1}{P_Y(A \mid p)}\,\lambda _2^{2} + \frac{1}{P_Y(a \mid p)}\,\lambda _2^{2} = \lambda _{2}^{2} \left( \frac{1}{P_Y(A \mid p)} + \frac{1}{P_Y(a \mid p)} \right) . \end{aligned}$$

Because the derivative of *each* output probability with respect to *p* is exactly $\pm \lambda _2$ (Eq. (9)), every effect of one generation of transmission on inferential precision is carried by the single scalar $\lambda _2$; this is why $\lambda _2$, rather than *a* and *b* individually, governs the Fisher Information analysis throughout the remainder of the paper.

Equation (10) contains the source Fisher Information, Eq. (8), as the noiseless special case $\lambda _2=1$ (i.e. a=1,b=0, so that $P_Y(A\mid p)=p$ and $P_Y(a\mid p)=1-p$): a perfectly faithful channel carries the full source Fisher Information forward unchanged. In Sect. 3 we specialize Eq. (10) to the noise-parametrized channel of Eq. (11), for which $\lambda _2=1-2\theta$, recovering Eq. (13) as a direct corollary rather than a separate assumption.

## Connection to the Quantum Channel Framework

A complementary perspective arises from Frieden’s information-theoretic formulation of physical law (Frieden 2024), in which observable structure emerges through the transfer of Fisher information from an underlying, pre-existing reservoir *J* to the physical system. In this framework, physical dynamics — including quantum wave-function formation — are shaped by the maximization of the net information flow J-I. The equivalence between the genetic transmission channel and the quantum depolarizing channel can therefore be viewed as a specific realization of this broader principle: both systems instantiate the same geometric constraint on how much Fisher information can be preserved as noise perturbs an initially structured state. This connection situates the genetic channel not merely as an analogue of a quantum process, but as an example of a general information-flow mechanism governing the emergence and degradation of organized biological configurations.

This classical genetic channel is formally equivalent to the quantum depolarizing channel. Parameterizing noise by $\theta \in [0,\tfrac{1}{2}]$, set

11 $$\begin{aligned} a = 1-\theta , \qquad b = \theta , \end{aligned}$$

so that, by Eq. (4), $\lambda _2 = 1-2\theta$.

For a Bernoulli input $X \sim \textrm{Bern}(p)$, the offspring success probability follows directly from Eq. (5) upon substituting Eq. (11):

12 $$\begin{aligned} y(p,\theta ) \equiv P_Y(A\mid p) = (1-\theta )p+\theta (1-p) = \theta + p(1-2\theta ), \end{aligned}$$

with $$P_Y(a\mid p) = 1-y(p,\theta )$$.

*The Fisher Information at the channel output* follows immediately as a corollary of the general two-state result, Eq. (10), derived in Sect. 2.2. Substituting $\lambda _2=1-2\theta$, $P_Y(A\mid p)=y$, and $P_Y(a\mid p)=1-y$ into Eq. (10) gives

13 $$\begin{aligned} I_{\textrm{out}}(p,\theta ) = (1-2\theta )^{2}\left( \frac{1}{y}+\frac{1}{1-y}\right) = \frac{(1-2\theta )^{2}}{y(1-y)}. \end{aligned}$$

In the noiseless limit (θ=0, so $\lambda _2=1$ and y=p), Eq. (13) reduces to the source Fisher Information already obtained in Eq. (8),

14 $$\begin{aligned} J(p) = \frac{1}{p(1-p)}. \end{aligned}$$

The fraction of information preserved under transmission is therefore

15 $$\begin{aligned} \frac{I_{\textrm{out}}}{J} = (1-2\theta )^{2}\,\frac{p(1-p)}{y(1-y)}, \end{aligned}$$

which decreases monotonically with noise and vanishes at $\theta =\tfrac{1}{2}$.

### Eigenvalue Control of Information Flow

Section 2.2 showed that, for *any* two-state channel with eigenvalues $\lambda _1=1$ and $\lambda _2$, the Fisher Information carried by the channel output about the parental frequency *p* takes the compact form of Eq. (10), with $P_Y(A \mid p)$ and $P_Y(a \mid p)$ given in Eqs. (5) and (6), and that Eq. (13) above is simply this compact form evaluated on the noise-parametrized channel of Eq. (11). Thus information decay is governed entirely by $\lambda _2$, and not by *a* and *b* individually: any two channels sharing the same $\lambda _2$ transmit identical Fisher Information, regardless of how their individual transition probabilities are composed. This is the sense in which $\lambda _2$ quantifies the loss of distinguishability between allelic states across generations, and it is why directional selection — which, as shown in Appendix A, acts by shifting $\lambda _2$ itself — is an effective lever for offsetting mutational noise regardless of the specific mutational mechanism generating that noise.

## Thresholds: Classical, Quantum, and Thermodynamic

### Fluctuation-Sensitive Collapse

Following the fluctuation-sensitive entropy functional (Glasenapp et al. 2015)

16 $$\begin{aligned} G(p) = \sqrt{p(1-p)} + 2p(1-p), \end{aligned}$$

the fluctuation amplitude

17 $$\begin{aligned} \eta = \sqrt{p(1-p)} \end{aligned}$$

plays a central role. In the equivalent quantum channel, entanglement vanishes at the Werner-state threshold η=1/3, implying

18 $$\begin{aligned} p(1-p) = \frac{1}{9}. \end{aligned}$$

Solving Eq. (18) yields

19 $$\begin{aligned} p_{\textrm{collapse}} = \frac{1+\sqrt{5/9}}{2} \approx 0.873. \end{aligned}$$

### Fisher Information at Collapse

From the collapse condition (Eq. (18)), the source Fisher Information at $p_{\textrm{collapse}}$ follows directly from Eq. (14):

20 $$\begin{aligned} J(p_{\textrm{collapse}}) = \frac{1}{p_{\textrm{collapse}}(1-p_{\textrm{collapse}})} = \frac{1}{1/9} = 9. \end{aligned}$$

At this threshold, the state-contrast function of Eq. (B3) — introduced here in preview and justified fully in Appendix B, where it is shown to represent the largest fraction of transmission fidelity retainable by any channel consistent with the state *p*, and is *not* the mutation-channel eigenvalue $\lambda _2$ of Sections 2–3 — takes the value

21 $$\begin{aligned} &  \kappa (p_{\textrm{collapse}})^{2} = \frac{5}{9}, \qquad I_{\textrm{out}} = \frac{5}{9}\,J(p_{\textrm{collapse}}) = \frac{5}{9}\cdot 9 = 5,\nonumber \\ &  \qquad I_{\textrm{loss}} = \frac{4}{9}\,J(p_{\textrm{collapse}}) = \frac{4}{9}\cdot 9 = 4. \end{aligned}$$

**Fig. 1:**
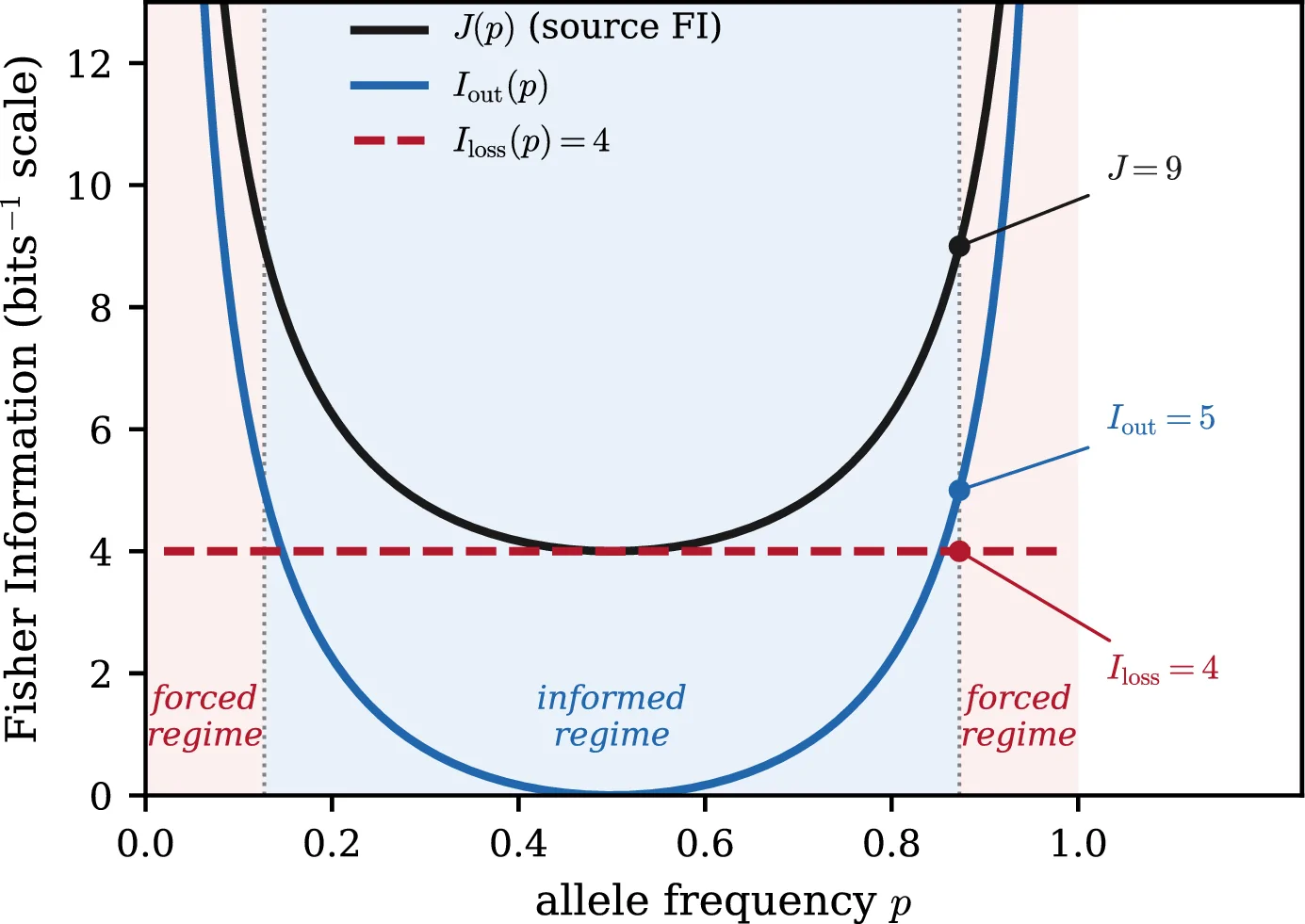
Decomposition of the source Fisher Information J(p)=1/[p(1-p)] (black) into the retained component $I_{\textrm{out}}(p)=\kappa (p)^{2}J(p)$ (blue) and the lost component $I_{\textrm{loss}}(p)=4$ (red, dashed), for the limiting channel of Appendix B.3 in which $\lambda _2=\kappa (p)$ (Eq. (23)). The lost component is constant across all *p*, equal to J(1/2)=4. Dotted vertical lines mark the instability boundary $p^{*}\approx 0.873$ and its mirror $1-p^{*}\approx 0.127$ (Eq. (19)), where the marked values reproduce Eq. (21). Shading distinguishes the informed regime (p(1-p)>1/9) from the forced regime (p(1-p)<1/9) of Appendix B.5

Thus

22 $$\begin{aligned} I_{\textrm{loss}} = 4 = J(0.5), \end{aligned}$$

the minimum Fisher Information of a maximally heterogeneous population. This is, in fact, not a coincidence restricted to $p_{\textrm{collapse}}$: for the limiting channel of Appendix B.3 ($\lambda _2=\kappa (p)$), writing $I_{\textrm{out}}(p) = \kappa (p)^{2}J(p)$ and $I_{\textrm{loss}}(p) = [1-\kappa (p)^{2}]J(p)$ and substituting $\kappa (p)^{2}=(2p-1)^{2}$ (Eq. (B3)) and J(p)=1/[p(1-p)] (Eq. (14)) gives

23 $$\begin{aligned} I_{\textrm{loss}}(p) = \bigl [1-(2p-1)^{2}\bigr ]\,\frac{1}{p(1-p)} = 4p(1-p)\cdot \frac{1}{p(1-p)} = 4 \end{aligned}$$

for *every* p∈(0,1), not only at $p_{\textrm{collapse}}$ or p=0.5: under this limiting channel, the information lost to depolarization is a constant, exactly equal to the minimum attainable source information *J*(1/2), while the retained information $I_{\textrm{out}}(p) = J(p) - 4$ grows without bound as *p* moves away from 1/2. Figure 1 illustrates this decomposition across the full range of *p*, showing $I_{\textrm{loss}}$’s constancy alongside the independently derived instability boundary of Eq. (19).

### Thermodynamic Interpretation of the Collapse Boundary

Independent thermodynamic analyses reveal that the same fluctuation boundary governs the energetic stability of fine-grained molecular states (Gatenby and Glasenapp 2026). In these models, the structural sensitivity depends on the variance-like term x=p(1-p), which reaches a consistent limit at

24 $$\begin{aligned} x = \frac{1}{9}. \end{aligned}$$

Strikingly, this thermodynamic boundary coincides exactly with the Fisher Information collapse derived from the quantum-channel representation (Eq. (18)) (Glasenapp 2025). The convergence of these results — one from channel geometry, one from energetic stability — suggests that the collapse at p≈0.873 reflects a shared constraint on the maintenance of structured biological information.

**Selection as an anti-depolarizing force.** In biological systems, selection pressure provides the counteracting force that maintains distinguishability against this intrinsic depolarizing drift — that is, a force that resists the channel’s tendency to drift toward the fully mixed, information-erasing state (the classical analogue of the quantum *depolarizing channel* introduced in Sect. 3). In the channel framework, selection effectively increases the magnitude of the nontrivial eigenvalue $\lambda _2$ (Eq. (4)), thereby enhancing information retention and resisting the homogenizing tendency of the symmetric circulant operator. A formal derivation of the selection-modified eigenvalue and its implications for information retention are provided in Appendix A; the geometric and algebraic origin of the 1/9 boundary itself is established in Appendix B. This interpretation aligns with the thermodynamic picture in which adaptive work offsets entropic relaxation: selection acts as the biological mechanism that preserves non-uniformity until the fluctuation boundary p(1-p)=1/9 (Eq. (18)) is reached, beyond which the selective mechanism considered here can no longer prevent collapse.

## Implications for Gene Regulatory Networks

The Fisher Information analysis identifies a collapse at p≈0.873 (Eq. (19)), where inferential precision vanishes. A structurally suggestive parallel appears in a quantum-circuit model of gene regulatory networks: in that model, the interpretability of the controlled-rotation (*c*-$R_y$) gate as a measure of regulatory interaction strength breaks down once a control gene’s activation frequency exceeds p≈0.854 ($\varphi _1 > 0.75\pi$), a “boundary effect” arising from non-monotonicity in the composition of two sequential rotation gates (Roman-Vicharra and Cai 2022). We emphasize that this is a specific numerical feature of that circuit implementation — identified as a limitation of the model, with its authors noting its impact on their own results is likely small — rather than an independently established empirical threshold for gene regulatory instability in general. The closeness of the two values (85.4% vs. 87.3%) is nonetheless a striking and currently unexplained numerical convergence between two independently motivated quantum-information treatments of genetic regulation, and we note it here as a target for further investigation rather than as confirmatory evidence for the boundary derived in this paper.

Independently of this parallel, quantum-channel analysis shows that entanglement collapses at the same fluctuation boundary p(1-p)=1/9 (Eq. (18)). Thermodynamic fine-graining models likewise identify this point as the limit at which the system undergoes a phase transition due to the failure of the energetic mechanism that maintains low-entropy structure.

The Fisher Information collapse, quantum decoherence, and thermodynamic fine-graining failure therefore appear not to be independent phenomena (Gatenby and Glasenapp 2026). They are consistent with manifestations of a shared information-geometric constraint that governs the stability of biological organization across scales. Within this framework, selection pressure can be understood as the biological force that counteracts channel depolarization, maintaining structure and regulatory fidelity against the natural drift toward uniformity. Its failure at the same fluctuation boundary p(1-p)=1/9 suggests that the collapse is not merely statistical or thermodynamic but reflects a shared limit on the capacity of selection to preserve information in the face of noise.

## Biological Expression of Fisher–Information Limits in Cancer Evolution

Carcinogenesis, the stepwise transformation of normal mammalian cells to a malignant phenotype with unconstrained proliferation, is often described as “somatic evolution”. This transition is typically associated with accumulating mutations (Rozhok and DeGregori 2015), and some carcinogenesis models posit a “mutator phenotype” (Loeb 2001) that permits an increased mutation rate as a necessary condition for the associated somatic evolution. The necessity of some minimum threshold of mutations and the requirement for a systematically increased mutation rate remain controversial (Gatenby and Brown 2017). Nevertheless, carcinogenesis in different species and different organisms converges (Bhattacharya et al. 2025) on a cellular phenotype and tissue organization with less structure and function than the tissue of origin, implying an increased entropy and loss of information (Tarabichi et al. 2013). Typically, the environment of cancer cells fluctuates due to an unstable vascular system in which blood flow is chaotic, resulting in both cycling and prolonged periods of hypoxia and acidosis (Gillies et al. 2018) with associated increased mutation rate (Xiao et al. 2003; Luoto et al. 2013).

Thus, carcinogenesis can be viewed as a transition in evolutionary selection on intracellular information dynamics (Brown et al. 2023). Normal mammalian cells contribute to and are defined by the fitness of the multicellular organism. Here, the evolutionary imperative is to maintain the existing cellular information state to maintain robust tissue function (Gatenby et al. 2020). In contrast, individual cancer cells can be viewed as single-cell protists (Gatenby et al. 2025) under evolutionary selection to maximize their proliferation within the local environmental conditions. This produces genotypic diversity within individual cells of a cancer population with corresponding degradation of tissue structure.

Viewed through this lens, carcinogenesis can be framed as more than a sequence of mutations: a shift from host-regulated, high-fidelity transmission to maintain tissue homeostasis, to a regime in which selection acts as an anti-depolarizing force, preserving only the information necessary to maintain intracellular regulatory structures required for withstanding intrinsic channel noise and harsh environmental conditions. The empirical patterns observed across cancers are consistent with, and offer biological support for, the central hypothesis of this work — that the stability, diversity, and regulatory precision of genetic systems are bounded by limits on information transmission.

Within the Fisher Information framework introduced here, this pattern emerges naturally as the expected behavior of a regulatory system approaching the instability boundary

25 $$\begin{aligned} p(1-p) = \frac{1}{9} \end{aligned}$$

(cf. Eq. (18)). As channel noise increases and the nontrivial eigenvalue $\lambda _2$ (Eq. (4)) decays, only those components of the regulatory architecture that directly contribute to survival and proliferation can be stably maintained. High-entropy, energetically costly differentiated functions collapse first, while low-entropy, fitness-enhancing modules persist or expand under selection. In this sense, the convergent cancer phenotype provides a biological instantiation of the same information-geometric constraint that governs genetic stability across the history of life. We note that this information connection of mutation rate and environmental heterogeneity suggests treatment strategies that generate a mismatch, such as enforcing a high mutation rate in cancers with a homogeneous environment. This might be observed, for example, in consistent cure of metastatic testicular cancers, which typically maintain an unusually homogeneous environment (and uniform cellular phenotype), with mutation-inducing platinum chemotherapy (Tateo et al. 2025; Lu et al. 2023; Schiantarelli et al. 2022). In contrast, cancers with a high mutation rate may be vulnerable to environmental stabilization and increased homogeneity — a strategy, termed “vascular normalization” or “microenvironmental normalization”, currently being explored (Choi and Jung 2023).

The environmental restructuring described above is a specific instance of a broader evolutionary phenomenon. Niche construction occurs when organisms alter their environment in ways that persist across generations and thereby enhance their own fitness — the textbook example being the beaver dam (Odling-Smee et al. 2003; Odling-Smee 2024). Because such modifications can outlast the individual and be inherited by descendants, niche construction has been proposed as an ecological mechanism of inheritance operating alongside genetic transmission (Laland et al. 2016). Cancer cells engage in niche construction most directly through angiogenesis, recruiting a vascular supply that persists and continues to support proliferation (Qian and Akçay 2018). Chronic inflammation, hypoxia, and acidosis are widely — though not invariably — observed features of the tumor microenvironment (Hanahan and Weinberg 2011; Nishida and Andoh 2025; Somarelli 2021), and while these are often attributed simply to inadequate or chaotic angiogenesis, an alternative view treats them as products of active niche construction that raises cancer cell fitness by promoting invasion and suppressing immune surveillance (Yang et al. 2014; Ibrahim-Hashim et al. 2017). We adopt this niche-construction framing as an interpretive hypothesis about *why* the tumor microenvironment takes the form it does, consistent with but not established by the information-geometric analysis above, and develop its informational analogue in Sect. 7.3.

## Discussion

The foregoing analysis establishes a unified information-geometric framework in which genetic stability, regulatory fidelity, and evolutionary adaptability are jointly constrained by the Fisher Information capacity of the transmission channel. Three biological implications of this framework merit explicit discussion, as they extend the formal results toward empirically testable predictions and connect the model to well-established concepts in evolutionary and cancer biology.

### Sustainability of the Mutation–Selection Equilibrium

A natural concern about any model in which selection only *partially* offsets mutation-driven depolarization is whether the resulting information loss is sustainable across generations. The framework resolves this concern precisely. At or near the ESS, the selective bias Δ is sufficient to hold the nontrivial eigenvalue $\lambda _2$ (Eq. (4)) well above the collapse threshold, so that the FI of the channel is maintained indefinitely. The phrase “partial offset” does not imply inexorable decay; it means that selection cannot neutralize *all* stochastic mutation-driven noise, leaving a small but bounded residual loss per generation. The long-run information state is a dynamic equilibrium, not a trajectory toward collapse.

This maps directly onto the classical drift–selection balance in population genetics (Dimijian 2012). For a selective advantage *s* and effective population size $N_e$, the criterion $N_e s \gg 1$ marks the regime in which selection dominates drift and allele frequencies are governed by adaptive fitness rather than random fluctuation. In our eigenvalue formulation, the selective bias Δ plays the role of *s*, stabilizing $\lambda _2$ above the critical threshold in the same way that $N_e s$ keeps alleles from drifting to fixation. The mutation rate θ (equivalently, the off-diagonal element *b* of the transition matrix in Eq. (3)) acts as drift: a depolarizing force that reduces $|\lambda _2|$. At equilibrium, the channel retains a stable, non-zero Fisher Information, just as drift–selection balance maintains stable polymorphism without fixation or erasure of variation.

Decay becomes catastrophic only under two conditions: (a) when θ rises substantially above the ESS baseline, or (b) when the fluctuation term p(1-p) approaches $\tfrac{1}{9}$ (Eq. (18)), the instability boundary established via the quantum Werner-state criterion and its geometric characterization (Sect. 4.1, Appendix B). Below this boundary, the equilibrium is sustainable indefinitely — an information-theoretic analogue of mutation–selection balance, in which a steady-state level of heritable variation is maintained by the interplay of new mutation and purifying selection.

### The Instability Boundary as a Joint Function of Mutation Rate and Selection

The instability threshold $p(1-p)=\tfrac{1}{9}$ (Eq. (18)) is formally a condition on the allele frequency *p*, but its biological meaning is best understood as a joint constraint on mutation rate and selection intensity. The two parameters enter asymmetrically.

The mutation rate θ governs the *speed* at which the system approaches the boundary: higher θ accelerates depolarization, driving *p* toward the collapse region more rapidly. Directional selection Δ, by contrast, determines *how far* from the boundary the system can be held. Because $\lambda _{2}^{\textrm{sel}} = \lambda _2 + 2\Delta$ (Appendix A, Eq. (A2)), a stronger selective force extends the informed regime by raising the maximum achievable $\lambda _2$ under row-stochastic constraints. The two parameters are therefore inversely coupled in the stability condition: a higher selection intensity can sustain a correspondingly higher mutation rate without crossing the collapse boundary.

This inverse coupling has a direct implication for cancer biology. Within this framework, the “safe” mutation rate for a somatic cell is not a fixed biological constant but a dynamic function of the current selection pressure in the tumor microenvironment. In a harsh, fluctuating environment characterized by hypoxia, acidosis, and immune predation, selection intensity is both high and heterogeneous. The model predicts that an elevated mutation rate is not merely tolerated under these conditions but is actively favored, because the strong anti-depolarizing force of intense selection extends the region of the Bernoulli manifold in which the cell’s essential regulatory program remains in the informed regime.

Species-specific and tissue-specific ESS mutation rates can therefore be understood as reflecting the local balance of these opposing forces (Wei et al. 2022; Lynch et al. 2023). Cancer cells that succeed in harsh microenvironments are, in effect, those that have dynamically re-tuned this balance toward simultaneously higher θ and higher Δ — a prediction consistent with the mutator phenotype hypothesis (Loeb 2001) and with empirical observations of elevated mutation rates in hypoxic and acidic tumor regions (Xiao et al. 2003; Luoto et al. 2013).

### Cellular Entropy State, Informational Tolerance, and Niche Construction in Cancer

The Fisher Information framework also provides a natural account of why cancer cells are more tolerant of elevated mutation rates than the normal cells from which they arise. A highly differentiated normal cell maintains a complex, energetically costly regulatory architecture — tissue-specific gene expression programs, morphological constraints, and intercellular signaling networks — that requires high FI fidelity to sustain. Its baseline fluctuation term p(1-p) is correspondingly low, placing it well within the informed regime with a generous margin before the collapse threshold (Eq. (18)).

A cancer cell, having shed much of that differentiated architecture, operates at a higher basal entropy. Critically, the information content of the regulatory state it must maintain is lower: the essential program reduces to the core modules required for individual cell survival and proliferation. The effective p(1-p) distance from the boundary is therefore not set by the cell’s allele frequency alone, but by the *informational load* of the regulatory program it must sustain. A structurally simpler program requires less Fisher Information, so the same channel noise that would be catastrophic for a differentiated cell is sub-threshold for a de-differentiated one. This is consistent with the thermodynamic argument developed in Sect. 4.3: the adaptive work required to maintain a functional configuration scales with its complexity, so de-differentiation effectively lowers the energetic cost of resisting entropic relaxation.

This reasoning points toward a further, and more provocative, extension of the model. By inducing chronic hypoxia, acidosis, and inflammation in the tumor microenvironment (Gillies et al. 2018; Kondo et al. 2001; Aubert et al. 2024), cancer cells effectively elevate the channel noise θ for *all* cells in that niche — including normal stromal cells, infiltrating immune cells, host epithelial cells adjacent to the tumor and exposed to diffusing acid (Gatenby and Gawlinski 1996), and potentially competing cancer stem-cell populations. For normal cells, whose regulatory architecture is complex and whose distance from the instability boundary is small, this imposed increase in noise may be sufficient to push critical regulatory subsystems (immune recognition, DNA damage response, tissue homeostasis signals) past $p(1-p) = \tfrac{1}{9}$ (Eq. (18)), causing functional collapse precisely in the subsystems that would otherwise constrain tumor growth. This is consistent with multiple studies showing hypoxia and acidosis suppress immune response to cancer cells (Damgaci et al. 2018) and is specifically associated with T cell exhaustion (Scharping et al. 2021). It is also consistent with the observation that elevated mutation rates and altered function accumulate in intratumoral lymphocytes (Mukohara et al. 2024; Koufaris et al. 2025; Olcina et al. 2018; Kleppe et al. 2015), tumor-associated endothelial cells (Hida and Klagsbrun 2005), and peritumoral normal tissue beyond the visible tumor margin (Redon et al. 2010; Aran et al. 2017). We emphasize that this body of evidence establishes *correlation* between tumor-driven microenvironmental stress and dysfunction in neighboring cell populations; it does not by itself establish the specific information-theoretic mechanism — elevation of channel noise θ past the collapse boundary in Eq. (18) — proposed here to explain it. That mechanism remains a testable hypothesis, for which we outline explicit predictions below.

This hypothesis constitutes a form of *evolutionary niche construction* operating at the information-theoretic level: cancer cells reshape the informational landscape of their microenvironment to preferentially destabilize the regulatory fidelity of cells whose function represents the principal selection pressure against them. Having already reduced their own regulatory complexity to the minimum required for proliferation, cancer cells are more tolerant of the same environmental perturbation they impose on their neighbors. This framework, therefore, offers a principled, mechanistic interpretation of the well-documented phenomenon by which tumors actively corrupt the surrounding tissue microenvironment (Bhattacharya et al. 2025; Tarabichi et al. 2013).

Formalizing this argument rigorously will require extending the current framework to include a cell-type-specific *information load* parameter, quantifying how much Fisher Information is required to maintain the full differentiated regulatory program versus a stripped-down survival program. Such an extension would allow explicit modeling of the differential tolerance to channel noise between normal and malignant cells, and would make the niche construction hypothesis directly testable: tumors that generate more extreme microenvironmental stress should show earlier and more pronounced collapse of the immune and stromal regulatory fidelity, with correspondingly accelerated progression. This represents a natural and important direction for further development of the model.

### Proposed Extension: Wright’s *F*-Statistics as Information-Geometric Population Structure

The framework developed here operates on a single, homogeneous transmission channel, treating the allele frequency *p* as a population-level parameter. A biologically important and formally natural extension is to hierarchical population structure, in which genetic information is partitioned across multiple levels simultaneously — individuals within subpopulations, subpopulations within a total population, or subclones within a tumor. We propose that Wright’s *F*-statistics provide the appropriate bridge between the current eigenvalue formulation and this richer setting, and we outline here why this connection is geometrically principled rather than merely analogical.

Wright’s *F*-statistics — $F_{IT}$, $F_{IS}$, and $F_{ST}$ — partition the total genetic variance of a population across hierarchical levels (Wright 1951). The fixation index $F_{ST}$ in particular measures the proportion of total genetic variance attributable to differences *between* subpopulations:

26 $$\begin{aligned} F_{ST} \;=\; \frac{\sigma ^{2}_{\bar{p}}}{\bar{p}(1-\bar{p})}, \end{aligned}$$

where $\bar{p}$ is the mean allele frequency across subpopulations and $\sigma ^{2}_{\bar{p}}$ is the variance of subpopulation allele frequencies around that mean. Both numerator and denominator of Eq. (26) are variances: the numerator is the observed between-subpopulation variance, and the denominator $\bar{p}(1-\bar{p})$ is the variance of a Bernoulli distribution at the mean frequency $\bar{p}$ — the same variance term, rather than the fluctuation amplitude $\eta =\sqrt{p(1-p)}$ (Eq. (17)), that appears throughout this paper. Because the source Fisher Information is $J(\bar{p})=1/[\bar{p}(1-\bar{p})]$ (Eq. (14)), $F_{ST}$ can be written exactly as

27 $$\begin{aligned} F_{ST} \;=\; \sigma ^{2}_{\bar{p}} \, J(\bar{p}), \end{aligned}$$

an information-weighted variance: the observed between-subpopulation variance, scaled by the local inferential sensitivity $J(\bar{p})$ at the population’s mean frequency. $F_{ST}$ is therefore already an information-theoretic quantity in disguise, and its connection to the present framework is via the same source Fisher Information *J*(*p*) used throughout Sect. 2, rather than via the fluctuation amplitude η or the state-contrast function κ(p) of Appendix B, both of which depend on the standard-deviation scale $\sqrt{p(1-p)}$ rather than the variance p(1-p) itself.

This observation motivates a direct translation into eigenvalue terms — with one scaling caveat carried over from the correction above. Because $F_{ST}$ is a variance ratio bounded on [0, 1], the natural eigenvalue counterpart is the squared eigenvalue $\lambda _2^{2}$ (itself bounded on [0, 1] and, per Eq. (10), the quantity that directly scales Fisher Information), rather than the bare eigenvalue $\lambda _2$, which is a contrast-scale quantity bounded on [-1,1] and not directly commensurate with a variance ratio. When $F_{ST}=0$, subpopulations are informationally indistinguishable from the total population: all allelic contrast is within-group, and the between-group channel operates as a fully depolarizing operator with $\lambda _2^{2} \approx 0$. When $F_{ST}=1$, subpopulations are completely differentiated, corresponding to $\lambda _2^{2}$ near its maximum contrast-preserving value of 1. The squared nontrivial eigenvalue $\lambda _2^{2}$ and the fixation index $F_{ST}$ are therefore proposed to measure a structurally similar quantity — the fraction of total variance, or total contrast, preserved across a transmission step — at different levels of a population hierarchy; Eq. (27) makes this precise for $F_{ST}$ itself, while the $\lambda _2^{2}$ correspondence remains, like the rest of this subsection, a proposed structural parallel rather than a derived identity.

Under this proposed mapping, the instability boundary $p(1-p)=\tfrac{1}{9}$ (Eq. (18)) acquires a natural hierarchical interpretation. It identifies the minimum $F_{ST}$ below which the regulatory fidelity of subpopulation structure cannot be maintained against depolarizing noise. When between-group contrast falls below this geometric threshold, the subpopulation structure collapses informationally: the channel can no longer sustain distinguishable allelic signals at the group level, regardless of the selective forces acting on individual subpopulations. This has immediate implications for three empirical domains.

In *conservation biology*, small and fragmented populations are known to lose within-population diversity and increase $F_{ST}$ through genetic drift and founder effects (Frankham 1996). The proposed framework predicts a specific collapse boundary: below a critical $F_{ST}$ determined by $\bar{p}(1-\bar{p}) = \tfrac{1}{9}$, population substructure becomes informationally unsustainable, and regulatory fidelity at the subpopulation level degrades irreversibly. This would provide a theoretically grounded threshold for minimum viable population structure, complementing existing $N_e$-based criteria.

In *tumor evolution*, intratumoral subclone dynamics represent a direct analogue of hierarchical population structure, with subclones playing the role of subpopulations and the tumor as the total population (Bhattacharya et al. 2025). High intratumoral $F_{ST}$ corresponds to strong clonal differentiation and preserved subclonal identity; low $F_{ST}$ corresponds to a homogenized, depolarized tumor in which no subclone maintains a distinct informational signature. The instability boundary then predicts the conditions under which clonal structure collapses and the tumor transitions to a regime of undifferentiated, high-entropy proliferation — precisely the convergent cancer phenotype described in Sect. 6. Furthermore, as noted above, cancer cells may exploit their greater distance from the informational collapse threshold by maintaining a mutagenic hypoxic, acidic microenvironment to increase mutations in infiltrating normal immune cells, thus crossing the instability boundary with loss of function. This hypothesis is supported by whole genome sequencing of tumor-infiltrating lymphocytes showing acquired mutations that altered function, leading to an “exhausted” phenotype (Mukohara et al. 2024; Kleppe et al. 2015).

A second formal extension concerns the role of genetic drift in small populations. In the current model, θ is the sole source of channel depolarization. In a hierarchically structured population, drift — operating through finite effective population size $N_e$ within each subpopulation — constitutes a second, independent depolarizing force. Incorporating drift as an additive contribution to the total channel noise would formalize the drift–selection analogy introduced in Sect. 7.1 and give it quantitative precision: the combined depolarizing pressure on $\lambda _2$ would be

28 $$\begin{aligned} \lambda _{2}^{\textrm{total}} \;\approx \; \lambda _{2}^{\textrm{mut}} \cdot \lambda _{2}^{\textrm{drift}}, \end{aligned}$$

where $\lambda _{2}^{\textrm{mut}} = 1-2\theta$ captures mutation-driven relaxation as in the main text (Eqs. (11)–(13)), and $\lambda _{2}^{\textrm{drift}}$ encodes the additional contrast loss per generation due to finite $N_e$. Under the Wright-Fisher model, $\lambda _{2}^{\textrm{drift}}$ takes the approximate form $1 - \tfrac{1}{2N_e}$ for large $N_e$, recovering the standard result that drift is negligible when $N_e \gg 1$ (Ewens 2004). The combined eigenvalue framework would then allow the instability boundary to be expressed in terms of the joint $(N_e,\theta ,\Delta )$ parameter space, unifying mutation pressure, drift, and selection into a single information-geometric stability condition.

We emphasize that the *F*-statistic mapping and the multiplicative drift formulation described here are proposed extensions requiring formal derivation, not results established by the present analysis. In particular, the multiplicative eigenvalue decomposition (Eq. (28)) is an approximation whose validity conditions — including independence of mutation and drift noise sources and the range of $N_e$ for which the linearization holds — will need to be established rigorously. Nevertheless, the structural alignment between Wright’s variance-partitioning framework and the Fisher Information geometry of the Bernoulli manifold is precise enough that the connection is unlikely to be coincidental. Developing it formally represents what we regard as the most mathematically natural direction for extending the current framework to biologically realistic population structures.

### Limitations of the Two-State Model

The framework developed in this paper rests on a deliberately minimal representation of genetic transmission: a single locus with two alleles, symmetric mutation, and a well-mixed, unstructured population. We adopt this representation because it is the simplest setting in which the channel eigenvalue $\lambda _2$, the Fisher Information of Eq. (10), and the quantum-channel equivalence of Sect. 3 can all be defined in closed form and connected explicitly. Real genetic systems depart from this minimal model in several ways, and it is worth stating plainly which features of our results depend on the two-state simplification and which are expected to generalize.

*Multi-allelic loci.* Most loci segregate more than two alleles. The Bernoulli manifold of Section B would be replaced by a higher-dimensional simplex, on which the Fisher–Rao metric is still well defined but the transition operator *C* is no longer a 2×2 circulant matrix with a single nontrivial eigenvalue; instead, a spectrum of nontrivial eigenvalues governs the decay of distinguishability among the multiple alleles, and the single scalar collapse condition p(1-p)=1/9 (Eq. (18)) would need to be replaced by a condition on this full spectrum. We expect the qualitative picture — a boundary beyond which no eigenvalue of the transition operator can sustain stable inference — to persist, but its precise location and the number of independent boundaries are open questions.

*Asymmetric mutation.* The channel matrix *C* of Eq. (3) assumes equal forward and reverse mutation rates (*a* and *b* shared by both rows). Real mutation processes are frequently asymmetric (e.g. transition/transversion bias, CpG hypermutability). An asymmetric channel is no longer circulant, and its two eigenvalues need not be real or have the simple form of Eq. (4); the quantum-channel equivalence of Sect. 3, which relies on the circulant structure to map the channel onto the depolarizing channel, would need a corresponding generalization (most naturally to an asymmetric or amplitude-damping quantum channel) before the collapse threshold could be re-derived in this setting.

*Epistasis and gene regulatory network coupling.* The model treats each locus as an independent two-state channel. Real genetic systems are coupled through epistatic interactions and gene regulatory networks, in which the fitness or regulatory consequence of an allele at one locus depends on the state of others. Section 5 draws a qualitative parallel between our collapse boundary and empirically reported instability thresholds in gene regulatory networks, but a first-principles extension of the present channel formalism to coupled loci — for instance, by replacing the scalar *p* with a joint distribution over multiple loci and studying the spectrum of the resulting higher-dimensional transition operator — has not been attempted here and would be needed to make the GRN connection more than qualitative.

*Population structure.* The model treats the population as a single, well-mixed unit characterized by one frequency *p*. Section 7.4 outlines, but does not formally derive, an extension to hierarchically structured populations via Wright’s *F*-statistics; as noted there, the multiplicative decomposition of $\lambda _2$ into mutation and drift components (Eq. (28)) is a proposed approximation rather than an established result.

Each of these simplifications is a natural target for future extension of the framework rather than a limitation specific to the collapse threshold itself: the four-way convergence documented in Appendix B depends only on the geometry of the Bernoulli manifold, which is the exact and correct description of a single, symmetric, biallelic, unstructured locus. Whether the same numerical boundary, or a structurally analogous one, governs the multi-allelic, asymmetric, epistatically-coupled, population-structured case is accordingly a hypothesis motivated by the present results rather than a conclusion established by them.

## Conclusion

By analyzing genetic inheritance through the geometry of Fisher Information, we show that the stability of biological information flow is governed by the nontrivial eigenvalue of the transmission operator (Eq. (4)). Directional selection modifies this eigenvalue linearly (Appendix A, Eq. (A2)), amplifying the contrast-preserving mode and counteracting the depolarizing drift induced by mutation.

These divergent informational and evolutionary dynamics are readily evident in carcinogenesis. The constituent cells of a multicellular organism are subject to the evolutionary imperative of maintaining normal tissue structure and function. Thus, there is strong evolutionary selection to maintain genetic stability through depolarization control. However, in cancers, evolutionary selection favors the individual cancer cells to maximize their fitness and, therefore, proliferation in non-physiological environments subject to significant temporal and spatial fluctuations. Here, increased channel noise in the form of a higher mutation rate is favored to maximize adaptation to diverse and often toxic environmental conditions. Despite the remarkably diverse species and tissue of origin in cancers, these selection forces lead to convergence of common cellular properties characterized by the cancer hallmarks (Bhattacharya et al. 2025; Gatenby et al. 2025; Hanahan and Weinberg 2011).

However, the eigenvalue formulation also reveals an upper bound: when the fluctuation term p(1-p) approaches the limit 1/9 (Eq. (18)), even the maximal eigenvalue achievable under row-stochastic constraints fails to sustain Fisher Information stability. This same boundary marks the collapse of quantum coherence in the equivalent depolarizing channel and the energetic limit of thermodynamic fine-graining models (Sect. 4.3), with a numerically close parallel also noted in a quantum-circuit model of gene regulatory networks (Sect. 5). These convergences suggest that the loss of predictability, regulatory fidelity, and structural organization in biological systems reflects a shared information-geometric constraint rather than independent phenomena.

Beyond the convergent cancer phenotype itself, the framework generates a specific, falsifiable hypothesis about the tumor microenvironment: because cancer cells operate farther from the instability boundary than the normal cells they displace, they may tolerate — and, we propose, actively construct — the mutagenic, acidic, hypoxic niche that drives informational collapse in neighboring stromal and immune cells (Sect. 7.3). This niche-construction mechanism is not established by the present analysis; it is offered as a testable extension, together with the associated predictions that tumors generating more extreme microenvironmental stress should show earlier and more pronounced collapse of immune and stromal regulatory fidelity, and that microenvironmental normalization should restore that fidelity in a manner tracking the model’s collapse boundary.

Within this framework, selection preserves structure only up to the instability boundary; beyond it, the model predicts that collapse follows. This perspective provides a candidate explanation for the sharp transitions observed in genetic regulation, evolutionary dynamics, and single-cell inference, and highlights Fisher Information as a natural metric for identifying the limits of biological organization.

## Figures and Tables

**Fig. 2:**
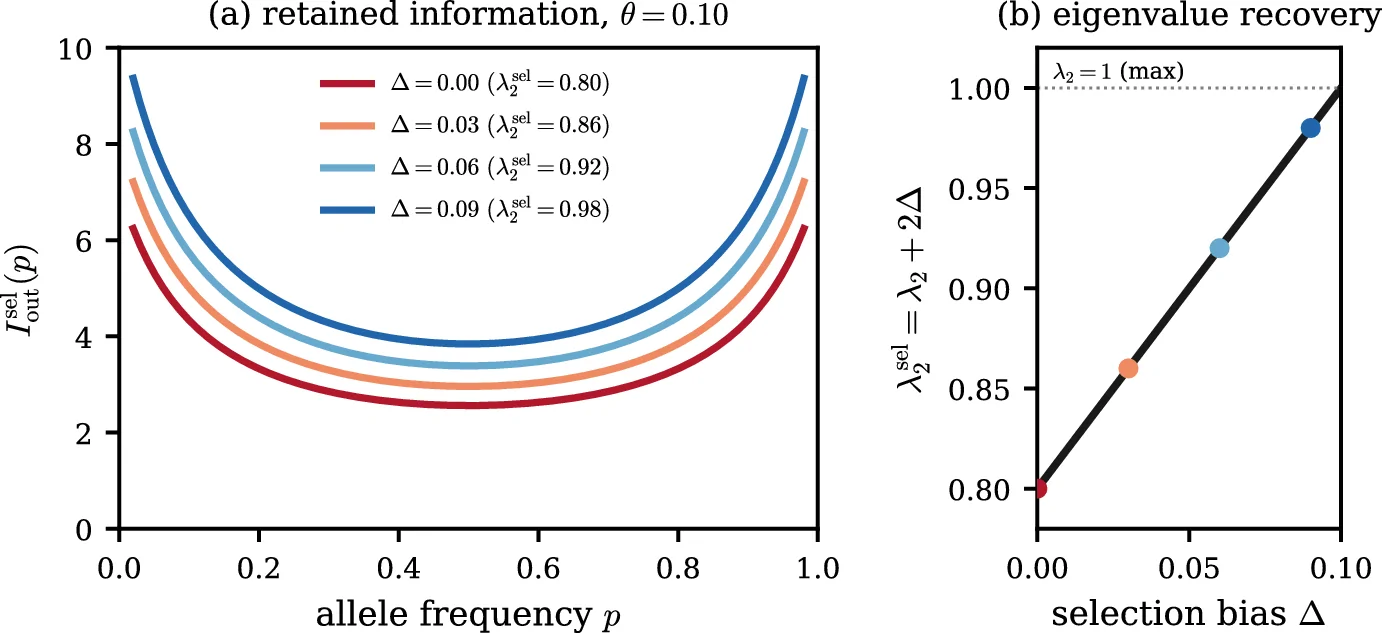
Selection-driven recovery of retained information for a channel with mutation rate θ=0.10. (a) $I_{\textrm{out}}^{\textrm{sel}}(p)$ (Eq. (A4)) for increasing selection bias Δ; larger Δ raises information retention uniformly across *p*. (b) The underlying mechanism is linear in Δ (Eq. (A3)), with Δ bounded above by $\min (a,b)=\theta$ (Eq. (A1)), so that $\lambda _2^{\textrm{sel}}$ can approach, but not exceed, the perfect-fidelity value $\lambda _2=1$ (Eq. (A5)). As Sect. b notes, this algebraic bound alone does not select the specific boundary $p(1-p)=\tfrac{1}{9}$; it establishes only the qualitative compensatory mechanism (color figure online)

## Appendix A: Selection as an Eigenvalue-Modifying Force in Genetic Information Channels

### Overview

In the main text, genetic transmission is modeled as a symmetric circulant channel (Eq. (3)) with eigenvalues $\lambda _1=1$ and $\lambda _2=a-b$ (Eq. (4)). The nontrivial eigenvalue $\lambda _2$ governs the decay of allelic contrast and therefore determines the Fisher Information retained across generations. Here, we formalize how selection pressure modifies this eigenvalue and acts as an anti-depolarizing force that counteracts entropic drift.

### Incorporating Selection into the Transition Operator

Selection acts as a *fidelity-enhancing* force: it suppresses the switching (mutational) probability *b* that drives depolarization, symmetrically with respect to the direction of switching, thereby increasing the diagonal (faithful-transmission) probability *a* by the same amount. Let Δ denote the magnitude of this selection-driven reduction in switching probability. The corresponding perturbed transition operator is

A1 $$\begin{aligned} C_{\textrm{sel}} = \begin{pmatrix} a+\Delta & b-\Delta \\ b-\Delta & a+\Delta \end{pmatrix}, \qquad 0 \le \Delta \le \min (a,b), \end{aligned}$$

which preserves both row-stochasticity and the symmetric circulant structure of Eq. (3): $C_{\textrm{sel}}$ is simply *C* with $a \rightarrow a+\Delta$ and $b \rightarrow b-\Delta$. This form is the natural minimal one, since it is the unique rank-one perturbation of *C* that (i) preserves row-stochasticity, (ii) preserves the $\mathbb {Z}_2$ symmetry of the channel (no directional bias toward *A* over *a* is introduced — selection here acts as *purifying* selection against switching events in either direction, not as directional selection favoring one allele), and (iii) therefore leaves the channel within the same symmetric circulant family analyzed in Sect. 2, so that Eqs. (4) and (10) apply to $C_{\textrm{sel}}$ without modification. An asymmetric perturbation of the off-diagonal terms (increasing b→A transmission while decreasing A→b transmission, or vice versa) would model directional selection favoring one allele, but — as verified explicitly via the trace and determinant of the resulting matrix in Sect. A.3 below — leaves the sum of the two off-diagonal transition probabilities, and hence the nontrivial eigenvalue $\lambda _2 = 1 - (\text {sum of off-diagonal probabilities})$, unchanged; such a perturbation shifts the *stationary frequency* toward one allele without altering the *rate* of information decay, and is therefore not the relevant mechanism for the fidelity question addressed here. We accordingly restrict attention to the fidelity-enhancing (rate-modifying) form of Eq. (A1).

### Effect on Eigenvalues

The eigenvalues of $C_{\textrm{sel}}$ follow from its trace and determinant. Writing $\tau = \textrm{tr}\,C_{\textrm{sel}} = 2(a+\Delta )$ and $\delta = \det C_{\textrm{sel}} = (a+\Delta )^{2}-(b-\Delta )^{2}$, the characteristic equation $\lambda ^{2}-\tau \lambda +\delta =0$ gives

A2 $$\begin{aligned} \lambda _{1}^{\textrm{sel}} = (a+\Delta )+(b-\Delta ) = 1, \qquad \lambda _{2}^{\textrm{sel}} = (a+\Delta )-(b-\Delta ) = (a-b) + 2\Delta , \end{aligned}$$

using a+b=1. Thus, selection acts *linearly* on the nontrivial eigenvalue:

A3 $$\begin{aligned} \lambda _{2}^{\textrm{sel}} = \lambda _2 + 2\Delta . \end{aligned}$$

(By contrast, for the asymmetric perturbation $$C'_{\textrm{sel}}=\bigl ({\begin{smallmatrix}a+\Delta & b-\Delta \\ b+\Delta & a-\Delta \end{smallmatrix}}\bigr )$$ considered in an earlier draft of this appendix, the trace is $\textrm{tr}\,C'_{\textrm{sel}}=2a$ and the determinant is $\det C'_{\textrm{sel}}=a^{2}-b^{2}$, both independent of Δ; the resulting eigenvalues are 1 and a-b, i.e. Δ cancels identically and directional selection of that form leaves $\lambda _2$ unchanged. This confirms that only the fidelity-enhancing form of Eq. (A1) produces the eigenvalue shift required for selection to act as an anti-depolarising force.)

Because Fisher Information scales as $\lambda _{2}^{2}$ (Eq. (10)), even small selective biases can substantially increase information retention:

A4 $$\begin{aligned} I_{\textrm{out}}^{\textrm{sel}}(p) = (\lambda _2 + 2\Delta )^{2} \left( \frac{1}{P_Y(A \mid p)} + \frac{1}{P_Y(a \mid p)} \right) . \end{aligned}$$

Selection therefore increases the distinguishability of allelic states by amplifying the contrast-preserving eigenmode of the channel. Figure  illustrates this effect: panel (a) shows $I_{\textrm{out}}^{\textrm{sel}}(p)$ for fixed mutation rate θ and increasing selection bias Δ, and panel (b) shows the underlying linear mechanism of Eq. (A3).

### Interpretation as an Anti-Depolarizing Force

In the absence of selection (Δ=0), the channel relaxes toward the fully depolarizing operator with $\lambda _2=0$, erasing all ancestral information. Selection counteracts this drift by increasing $|\lambda _2|$, thereby injecting structure (negentropy) into the system.

This interpretation aligns with the thermodynamic analyses of Sect. 4.3, in which adaptive work offsets entropic relaxation. In particular, the fine-graining framework identifies a collapse boundary at p(1-p)=1/9 (Eq. (18)), beyond which the energy cost of maintaining information exceeds the capacity of the biological channel. The eigenvalue formulation (Eq. (A2)) establishes the qualitative mechanism — selection shifts $\lambda _2$ linearly and thereby offsets mutational depolarization — but, as discussed in Sect. b below, the algebraic bound on Δ alone does not select the specific value $p(1-p)=\tfrac{1}{9}$; that value is established independently via the geometric and quantum-channel arguments of Sect. 4.1 and Appendix B.

### Bounds on Selection-Driven Eigenvalue Recovery

Because Δ is bounded by the requirement that transition probabilities remain non-negative, $0 \le \Delta \le \min (a,b)$ (Eq. (A1)), there exists a maximal eigenvalue achievable purely from suppressing the current channel’s own switching probability *b*:

A5 $$\begin{aligned} \lambda _{2,\max }^{\textrm{sel}} = (a-b) + 2\min (a,b) = a+b = 1. \end{aligned}$$

This bound is *p*-independent and, taken alone, is not the origin of the $p(1-p)=\tfrac{1}{9}$ boundary: it states only that, absent any further restriction on Δ, sufficiently strong purifying selection can in principle drive any mutation channel toward perfect fidelity ($\lambda _2\rightarrow 1$) by suppressing b→0. Equation (A5) therefore establishes that the eigenvalue-shift mechanism of Eq. (A3) is not bounded away from $\lambda _2=1$ by channel algebra alone; any *p*-dependent ceiling on the information a selection-modified channel can sustain must instead come from a constraint that ties Δ, or the eigenvalue itself, to the fluctuation state p(1-p) directly. Appendix B supplies this constraint in a different, purely geometric quantity — the state-contrast function κ(p)=2p-1, defined in Eq. (B3) — and Sect. b makes explicit that κ(p) is *not* the mutation-channel eigenvalue $\lambda _2^{\textrm{sel}}$ of the present appendix, but a property of the allele-frequency state itself. The two quantities coincide only in the limiting, purely illustrative case discussed in Sect. b, in which a channel’s transition probabilities are set equal to the population’s own current frequency (a=p,b=1-p); this limiting case is not implied by, or derivable from, the selection formalism of the present appendix, and the 1/9 threshold itself is imported from the quantum Werner-state criterion (Sect. 4.1) rather than derived from Eq. (A5).

This distinction matters for how the results of this appendix should be read: selection pressure demonstrably shifts $\lambda _2$ linearly (Eq. (A3)), which is the qualitative mechanism by which selection offsets mutational drift; but the specific numerical location of the instability boundary at $p(1-p)=\tfrac{1}{9}$ is established independently, in Sect. 4.1 and Appendix B, and should not be read as a consequence of the eigenvalue bound derived here.

### Conclusion of Appendix A

Selection pressure modifies the nontrivial eigenvalue of the genetic transmission channel (Eq. (A2)), acting as a stabilizing force that counteracts channel depolarization, preserving allelic contrast and information retention. This appendix establishes the mechanism by which selection counteracts mutational depolarization, and shows that the algebraic bound on the selection parameter Δ alone (Eq. (A5)) does not, by itself, fix the numerical location of an instability boundary. The fluctuation boundary p(1-p)=1/9 is established independently in Sect. 4.1 via the quantum Werner-state criterion and characterized geometrically in Appendix B; together, the mechanism of this appendix and the boundary value established elsewhere provide a unified account of the convergence of Fisher Information collapse, quantum decoherence (Sect. 3), and thermodynamic fine-graining failure (Sect. 4.3), with a numerically close parallel also noted in a quantum-circuit model of gene regulatory networks (Sect. 5).

## Appendix B: Geometric Characterization of the Instability Boundary

### Overview

The main text identifies the fluctuation threshold $p(1-p)=\tfrac{1}{9}$ (Eq. (18)) as the point at which Fisher Information collapse, quantum decoherence (Sect. 3), and thermodynamic fine-graining failure (Sect. 4.3) converge, with a numerically close parallel also noted in a quantum-circuit model of gene regulatory networks (Sect. 5). Appendix A establishes the mechanism by which selection offsets mutational depolarization, and shows that this mechanism alone does not fix a *p*-dependent stability ceiling. The present appendix supplies that ceiling geometrically, and addresses a complementary question: *what is the geometric and algebraic origin of the specific value 1/9?*

Although a purely classical derivation of this threshold from channel-capacity principles alone remains an open problem, the boundary is overdetermined in a precise sense: four independent characterizations, each approaching the problem from a distinct mathematical direction, converge on $p(1-p)=\tfrac{1}{9}$ as a qualitative boundary between two structurally distinct regimes. This convergence is not coincidental: as shown below, the threshold reflects the intrinsic geometry of the Bernoulli statistical manifold, independent of any particular biological interpretation.

### The Bernoulli Manifold and the Fisher–Rao Metric

The Bernoulli family provides the natural parameter space for the two-state genetic channel developed in Sect. 2. For a Bernoulli distribution with success probability *p*, the Fisher information is

B1 $$\begin{aligned} I(p) = \frac{1}{p(1-p)}, \end{aligned}$$

which defines a Riemannian metric — the Fisher–Rao metric — on the one-dimensional statistical manifold parameterized by p∈(0,1). This metric governs the curvature of the manifold and therefore the geometric cost of distinguishing nearby probability distributions.

A key property of this manifold is the role of coordinate systems in revealing its structure. The natural (logit) parameter $\eta = \log \!\bigl [\tfrac{p}{1-p}\bigr ]$ does not flatten the metric: in logit coordinates, the Fisher metric takes the form

B2 $$\begin{aligned} g_{\eta \eta } = p(1-p), \end{aligned}$$

precisely the variance-like term that appears throughout the main text. The flat coordinate — the one in which the metric is identically unity and geodesics are straight lines — is the arcsine transformation $\vartheta = 2\arcsin \!\sqrt{p}$. The quantity p(1-p) is therefore not an arbitrary variance term but the Fisher metric evaluated in the natural coordinate of the exponential family: it directly encodes the manifold’s curvature as seen from the most analytically convenient coordinate system. The threshold $p(1-p)=\tfrac{1}{9}$ (Eq. (18)) is accordingly a threshold on the manifold’s curvature, not merely on a statistical moment.

### The State-Contrast Function κ(p)

The four characterizations below make repeated use of the quantity $(2p-1)^{2}$. To avoid conflating this with the channel eigenvalue $\lambda _2$ of Sections 2 and 3, and Appendix A (which is a property of the *transmission operator* *C*, governed by the mutation parameter θ via $\lambda _2=1-2\theta$), we introduce distinct notation for it here. Define the *state-contrast function*

B3 $$\begin{aligned} \kappa (p) \equiv p - (1-p) = 2p-1, \end{aligned}$$

the difference between the two Bernoulli outcome probabilities themselves. Unlike $\lambda _2$, κ(p) is a property of the allele-frequency *state* *p*, not of any transmission channel acting on it: it requires no mutation parameter θ and no selection parameter Δ to define, and is well-defined even for a population considered in isolation, with no transmission step involved at all.

The two quantities coincide in exactly one limiting case: if a channel’s own transition probabilities are set equal to the current state, a=p,b=1-p, then by Eq. (4), $\lambda _2 = a-b = 2p-1 = \kappa (p)$. This limiting channel — in which the transmission operator simply reproduces the population’s own current frequency each generation, with no independent mutation or selection process — is a purely formal construction used below to give $\kappa (p)^2$ a channel-theoretic interpretation (Sect. b); it is *not* a claim that the mutation channel of Sect. 2 or the selection-modified channel of Appendix A takes this form, nor is κ(p) derived from the selection bound of Eq. (A5). With this notation fixed, we now give the four characterizations of the threshold in terms of κ(p).

### Four Characterizations of the Threshold

#### Geometric Characterization

The Fisher–Rao volume element on the Bernoulli manifold is

B4 $$\begin{aligned} \det g = \frac{1}{p(1-p)}. \end{aligned}$$

At the critical point $p^{*} \approx 0.873$, where $p^{*}(1-p^{*})=\tfrac{1}{9}$,

B5 $$\begin{aligned} \det g^{*} = 9. \end{aligned}$$

This is $\bigl (\tfrac{3}{2}\bigr )^{2}$ times the maximum-entropy baseline $\det g|_{p=1/2}=4$: the informational volume of the manifold is inflated by a factor of 9/4 relative to the reference state of maximum heterozygosity. Simultaneously, the state-contrast function of Eq. (B3) grows quadratically in *p*, $\kappa (p)^{2} = (2p-1)^{2}$. Beyond $p^{*}$, the geometric amplification of the manifold’s curvature grows faster than $\kappa (p)^{2}$ can compensate — the channel enters what may be termed the *forced regime*, in which the geometric cost of maintaining allelic distinguishability exceeds the transmission capacity available to *any* channel consistent with that state, including the limiting maximally-informative channel of Sect. B.3.

#### Algebraic Characterization

At the threshold (Eq. (18)), the state-contrast function satisfies

B6 $$\begin{aligned} \kappa (p^{*})^{2} = (2p^{*}-1)^{2} = 1 - 4p^{*}(1-p^{*}) = 1 - \frac{4}{9} = \frac{5}{9}. \end{aligned}$$

By the limiting-channel identity of Sect. B.3 ($\lambda _2=\kappa (p)$ when a=p,b=1-p), this is also the largest fraction of transmission fidelity retainable by any channel whose transition probabilities are set to reproduce the state *p* itself: at most 5/9, with the complementary fraction

B7 $$\begin{aligned} 1 - \kappa (p^{*})^{2} = \frac{4}{9} = 4\,p^{*}(1-p^{*}) \end{aligned}$$

lost to depolarization. The threshold is the unique interior point on (0, 1) at which retained and lost fidelity, in this limiting-channel sense, stand in the ratio 5 : 4. This result is consistent with the information-loss calculation in Sect. 4.2, where $I_{\textrm{loss}} = \tfrac{4}{9} J(p)$ at collapse (Eq. (21)).

#### Structural Characterization

Define the fluctuation amplitude $\eta (p) = \sqrt{p(1-p)}$ (Eq. (17)), which appears in the fluctuation-sensitive entropy functional *G*(*p*) (Eq. (16)). At the threshold,

B8 $$\begin{aligned} \eta ^{*} = \sqrt{p^{*}(1-p^{*})} = \sqrt{\frac{1}{9}} = \frac{1}{3}. \end{aligned}$$

Since η attains its maximum of $\tfrac{1}{2}$ at $p=\tfrac{1}{2}$, the value $\eta ^{*}=1/3$ is the point at which the allele-frequency standard deviation equals exactly two-thirds of its maximum. This is also the value at which the Werner-state entanglement criterion is saturated in the equivalent quantum depolarizing channel (Glasenapp 2025), providing the direct link between the classical genetic threshold and the quantum coherence boundary noted in Sect. 3.

#### Statistical Characterization

For a $\textrm{Bernoulli}(p)$ distribution, define the fluctuation ratio

B9 $$\begin{aligned} \Psi (p) = \frac{\textrm{SD}}{\textrm{Var}} = \frac{\sqrt{p(1-p)}}{p(1-p)} = \frac{1}{\sqrt{p(1-p)}}. \end{aligned}$$

This ratio is strictly increasing on (1/2, 1). At the critical threshold,

B10 $$\begin{aligned} \Psi (p^{*}) = \frac{1}{\sqrt{1/9}} = 3. \end{aligned}$$

For $p < p^{*}$, Ψ<3 and variance dominates fluctuations; for $p > p^{*}$, Ψ>3 and fluctuations are relatively amplified. The threshold is therefore the boundary at which the fluctuation ratio crosses the natural integer value 3, marking a transition from a variance-dominated to a fluctuation-dominated statistical regime.

### The Two Regimes

Together, the four characterizations support the identification of $p(1-p)=\tfrac{1}{9}$ as the boundary between two qualitatively distinct regimes:

- **The informed regime** ($p < p^{*}$): Fisher information, the state-contrast function κ(p), and the fluctuation measure jointly indicate that a stable informational signal remains achievable. The manifold’s curvature, though increasing, remains within the compensatory capacity available to the limiting maximally-informative channel of Sect. B.3. The system retains predictability and regulatory precision.
- **The forced regime** ($p > p^{*}$): The combined effect of geometric amplification, depolarization, and stochastic fluctuations exceeds the capacity even of that limiting channel, so no channel consistent with the state *p* — however strongly selected, per the mechanism of Appendix A — can sustain Fisher Information stability. Collapse — of Fisher Information, of quantum coherence, and of regulatory fidelity — follows.

This boundary is not a parameter chosen to fit biological data. It emerges from the intrinsic geometry of the Bernoulli manifold, independent of the biological context in which the channel is embedded. Its appearance across Fisher Information analysis (Sect. 4.2), thermodynamic fine-graining models (Sect. 4.3), and quantum channel theory (Sect. 3) reflects the fact that these three frameworks share the same underlying information geometry, with the numerically close parallel in gene regulatory network inference (Sect. 5) noted as a further, currently unconfirmed, point of resemblance.

### Open Problem

A rigorous derivation of the threshold from classical channel-capacity principles alone — without appeal to the quantum depolarizing channel analogy or the thermodynamic fine-graining argument — remains an open problem and represents a natural direction for future work. The four characterizations presented here establish the threshold’s geometric and algebraic necessity; a capacity-theoretic derivation would complete the information-theoretic picture.
